# Philadelphia Hospital—Women’s Lunatic Asylum

**Published:** 1844-05-04

**Authors:** 


### PHILADELPHIA HOSPITAL—WOMEN'S LUNATIC ASYLUM.

*Patients admitted from January* 1*st*, 1842, *to December* 31*st*, 1843, (*inclusive*,)

| American Paupers | Penn. | Del. | Md. | Va. | La. | N. Y. | N. J. | S. C. | Total. | SOCIAL STATE. Married. | Widow. | Single. | Total. |
|---|---|---|---|---|---|---|---|---|---|---|---|---|---|
| White, | 60 | 1 | 2 | 0 | 0 | 3 | 4 | 1 | 71 | 30 | 21 | 20 | 71 |
| Black, | 0 | 13 | 3 | 5 | 1 | 0 | 0 | 0 | 22 | 5 | 6 | 11 | 22 |

| Foreign Paupers. | Irel'd. | Ger'y. | Eng. | Scotl. | Wales | Africa. | France. | W. Ind. | Total. | SOCIAL STATE. Married. | Widow. | Single. | Total. |
|---|---|---|---|---|---|---|---|---|---|---|---|---|---|
| White, | 54 | 8 | 7 | 2 | 1 | 0 | 1 | 0 | 73 | 30 | 22 | 21 | 73 |
| Black, | 0 | 0 | 0 | 0 | 0 | 1 | 0 | 0 | 1 | 0 | 0 | 1 | 1 |

| | |
|---|---|
| Admitted, - - - - - - - - - - - - - | 167 |
| Of which were Delirium Tremens, - - - - - - - - | 31 |
| | 136 |
| Discharged, - - - - - - - - - - - - | 40 |
| | 96 |
| Died, - - - - - - - - - - - - - - | 11 |
| Remaining, - - - - - - - - - - - - | 85 |

*From January* 1*st to December* 31*st*, 1843.

| American Paupers. | Penn. | Del. | Md. | Va. | La. | N. Y. | N. J. | S. C. | Total. | SOCIAL STATE. Married. | Widow. | Single. | Total. |
|---|---|---|---|---|---|---|---|---|---|---|---|---|---|
| White, | 48 | 11 | 2 | 1 | 0 | 2 | 3 | 1 | 68 | 26 | 25 | 17 | 68 |
| Black, | 1 | 0 | 0 | 0 | 0 | 0 | 1 | 0 | 2 | 1 | 0 | 1 | 2 |

| Foreign Paupers. | Irel'd. | Ger'y. | Eng. | Scotl. | Wales | France. | W. Ind. | Africa. | Total | SOCIAL STATE. Married. | Widow. | Single. | Total. |
|---|---|---|---|---|---|---|---|---|---|---|---|---|---|
| White, | 47 | 2 | 3 | 3 | 3 | 1 | 0 | 0 | 59 | 24 | 20 | 15 | 59 |
| Black, | 0 | 0 | 0 | 0 | 0 | 0 | 0 | 1 | 1 | 1 | 0 | 0 | 1 |

| | |
|---|---|
| Remaining in Asylum January 1st, 1843, - - - - - - - | 85 |
| Admitted during the year 1843, - - - - - - - - | 130 |
| | 215 |
| Of which were Delirium Tremens, - - - - - - - - - | 28 |
| | 187 |
| Discharged, - - - - - - - - - - - | 72 |
| | 115 |
| Died, - - - - - - - - - - - - - | 14 |
| Remaining in the Asylum on the 31st of December, 1843, - - | 101 |

